# Epicardial adipose tissue thickness and plasma homocysteine in patients with metabolic syndrome and normal coronary arteries

**DOI:** 10.1186/1758-5996-6-62

**Published:** 2014-05-26

**Authors:** Akif Serhat Balcioğlu, Murtaza Emre Durakoğlugil, Davran Çiçek, Uğur Abbas Bal, Bülent Boyaci, Haldun Müderrisoğlu

**Affiliations:** 1 Department of Cardiology, Medical and Research Center of Alanya, Başkent University, Saray Mh. Yunus Emre Cad. No:1, 07400, Alanya, Antalya, Turkey; 2 Department of Cardiology, Faculty of Medicine, Recep Tayyip Erdoğan University, Rize, Turkey; 3 Department of Cardiology, Faculty of Medicine, Başkent University, Ankara, Turkey; 4 Department of Cardiology, Faculty of Medicine, Gazi University, Ankara, Turkey

**Keywords:** Angina pectoris, Coronary angiography, Epicardial fat, Homocysteine

## Abstract

**Background:**

Increased epicardial adipose tissue thickness and plasma homocysteine levels are associated with Metabolic Syndrome (MS) and coronary artery disease. The majority of patients with MS have subclinical or manifest coronary artery disease. The aim of this study was to evaluate the relationship between MS and plasma homocysteine levels and epicardial adipose tissue thickness in subjects without epicardial coronary artery disease.

**Methods:**

Patients who underwent coronary angiography due to angina or equivocal symptoms and/or abnormal stress test results and were found to have normal coronary arteries were evaluated for the presence of MS. The study group comprised 75 patients with normal coronary arteries and MS, and the control group included 75 age-gender matched subjects without coronary artery disease or MS.

**Results:**

Epicardial adipose tissue thickness (5.8 ± 1.9 mm vs. 4.3 ± 1.6 mm, p <0.001) and plasma homocysteine levels (21.6 ± 6.1 μmol/L vs. 15.1 ± 5.8 μmol/L, p <0.001) were significantly higher in the MS group. Body mass index, triglyceride level, weight, age and waist circumference were positively and HDL cholesterol level were negatively correlated with both epicardial adipose tissue thickness and plasma homocysteine level. Epicardial adipose tissue thickness had the strongest correlation with plasma homocysteine level (r = 0.584, p < 0.001). For each 1 mm increase in epicardial adipose tissue thickness, an increase of 3.51 μmol/L (95% CI: 2.24-4.79) in plasma homocysteine level was expected.

**Conclusions:**

We observed a close relationship between MS and epicardial adipose tissue thickness and plasma homocysteine levels, even in the absence of overt coronary artery disease.

## Background

Metabolic syndrome (MS) is a combination of disorders that increase the risk of cardiovascular disease and diabetes, including abdominal obesity, raised fasting plasma glucose, atherogenic dyslipidemia and high blood pressure [1]. Approximately 20 to 25 percent of the global population has MS, which is associated with a twofold increase in cardiovascular mortality and a threefold increase in the risk of myocardial infarction or stroke. In addition, the risk of developing type 2 diabetes is five times higher in people with MS [2]. Abdominal obesity and insulin resistance play a key role in the development of MS and data suggests that prothrombotic and proinflammatory states are also involved [3].

Homocysteine, a sulfur-containing amino acid, is produced through the metabolism of methionine. Elevated plasma homocysteine is associated with cardiovascular disease and stroke [4]. Studies have demonstrated that homocysteine induces endothelial cell injury and impairs endothelium-dependent vasodilation by decreasing the generation of nitric oxide and increasing the inactivation of nitric oxide [5-7]. The relationship between increased plasma homocysteine level and insulin resistance/hyperinsulinemia has been previously demonstrated [8,9]. In addition, higher plasma homocysteine concentrations have been detected in patients with MS and clinically manifest atherosclerotic vascular disease [10].

Epicardial adipose tissue (EAT), deriving from the same embryological origin as mesenteric and omental fat, is deposited around the heart, particularly around the coronary vessels [11]. EAT may influence coronary atherosclerosis and myocardial function due to the lack of a fibrous barrier blocking diffusion of free fatty acids and adipokines from the EAT to the underlying vessels and myocardium [11]. A recent meta-analysis demonstrated significantly higher echocardiographic EAT thickness in patients with MS [12]. EAT thickness is also related to epicardial obstructive coronary artery disease, myocardial ischemia, major adverse cardiac events and subclinical coronary artery disease [13-16].

It is known that the majority of patients with MS have subclinical or manifest coronary artery disease. As both plasma homocysteine and EAT are associated with coronary artery disease, the presence of concomitant coronary artery disease may influence the level of plasma homocysteine and EAT thickness in patients with MS. Therefore, the aim of this study was to evaluate the relation between MS and plasma homocysteine level and EAT thickness in subjects without epicardial coronary artery disease.

## Methods

Patients who underwent coronary angiography due to angina or equivocal symptoms and/or abnormal stress tests and who were found to have normal coronary arteries were evaluated for the presence of MS according to International Diabetes Federation criteria [17].

The study group comprised of 75 patients without coronary artery disease diagnosed with MS and the control group included 75 age-gender matched subjects without coronary artery disease or MS. The anthropometric characteristics of the control group were similar to those of the study group. All subjects in the control group had a risk factor of 0 or 1 for MS, mostly enlarged waist circumference (Table 1). The International Diabetes Federation defines MS as central obesity plus any two of the following four factors: raised fasting plasma glucose (≥100 mg/dL) or treatment of previously diagnosed type 2 diabetes, raised blood pressure (systolic blood pressure ≥130 or diastolic blood pressure ≥85 mmHg) or treatment of previously diagnosed hypertension, raised triglycerides (≥150 mg/dl), and reduced HDL cholesterol (<40 mg/dL in males, <50 mg/dL in females). Central obesity was accepted as a waist circumference of ≥94 cm in males and ≥80 cm in females in our study group [17]. Subjects with coronary artery disease, diabetes mellitus, folic acid deficiency, malabsorption syndromes, hypo-hyperthyroidism or chronic systemic illness were excluded. Patients using drugs that can affect the level of plasma homocysteine and those with a poor acoustic window were also excluded. The study was performed in accordance with the principles stated in the Declaration of Helsinki and was approved by the Medical Ethics Committee of Başkent University. All subjects gave informed consent prior to enrollment.

**Table 1:** Comparison of characteristics, blood samples and echocardiographic findings

	**Metabolic syndrome group (n = 75)**	**Control group (n = 75)**	**P value**
Age, years	59.6 ± 14.0	57.0 ± 15.2	NS
Gender, male/female, numbers (%)	28/47 (37.3/62.7)	32/43 (42.7/57.3)	NS
Hypertension, numbers (%)	22 (29.3)	14 (18.7)	NS
Height, cm	165.3 ± 8.5	164.9 ± 6.8	NS
Weight, kg	79.2 ± 10.3	73.5 ± 11.1	0.001
Body mass index, kg/m^2^	29.0 ± 3.6	27.1 ± 3.8	<0.001
Waist circumference, cm	93.8 ± 7.9	89.0 ± 9.6	<0.001
Central obesity criterion, numbers (%)	75 (100)	50 (66.7)	<0.001
Fasting plasma glucose, mg/dL	101.5 ± 12.1	99.5 ± 11.1	NS
Total cholesterol, mg/dL	191.4 ± 47.0	184.1 ± 43.5	NS
HDL cholesterol, mg/dL	40.4 ± 8.1	48.2 ± 11.1	<0.001
LDL cholesterol, mg/dL	116.5 ± 41.1	112.2 ± 38.1	NS
Trygliceride, mg/dL	172.4 ± 63.2	119.0 ± 53.0	<0.001
Systolic blood pressure, mmHg	128.5 ± 8.7	124.0 ± 10.7	0.001
Diastolic blood pressure, mmHg	80.9 ± 8.5	78.5 ± 7.1	0.021
Smokers, numbers (%)	18 (24)	23 (30.7)	NS
EAT thickness, mm	5.8 ± 1.9	4.3 ± 1.6	<0.001
Plasma homocysteine level, μmol/L	21.6 ± 6.1	15.1 ± 5.8	<0.001
Ventricular septum thickness, mm	11.5 ± 1.9	11.3 ± 1.6	NS
Posterior wall thickness, mm	10.6 ± 1.7	10.6 ± 1.5	NS
Left ventricular mass, g	185.4 ± 58.0	174.5 ± 46.9	NS
Left atrial diameter, mm	35.4 ± 5.2	35.3 ± 4.4	NS

Data are expressed as mean ± standard deviation or numbers (percentages). EAT = epicardial adipose tissue, NS = not significant.

Venous blood samples were collected after fasting for a minimum of twelve hours. Thyroid function, plasma glucose, total cholesterol, HDL cholesterol, LDL cholesterol, triglyceride, folic acid and homocysteine were measured. Plasma homocysteine assay was performed using the AxSYM immunoassay according to the manufacturer’s instructions. Plasma homocysteine levels between 3.36 and 20.44 μmol/L in men and 5.9 and 16 μmol/L in women were considered normal according to the kit used in the study.

Anthropometric measurements were obtained by well-trained medical staff using the same methods and instruments for all participant. Waist circumference was measured at the midpoint between the lower rib margin and the iliac crest.

Blood pressure values were obtained in the echocardiography room using the traditional auscultatory method with a sphygmomanometer. Patients were advised to refrain from smoking, consuming coffee or tea and physical exercise for 30 minutes and were seated for rest for five minutes prior to the measurement. Three separate measurements were averaged to determine office blood pressure.

Subjects underwent transthoracic echocardiography in the left lateral decubitus position using a General Electric Vivid e (General Electric HealthCare Ultrasound Cardiology, Horton, Norway) and a 3.5 MHz transducer according to the guidelines of the American Society of Echocardiography by the same cardiologist specialized in echocardiography [18]. EAT thickness was measured on the free wall of the right ventricle at the end-diastole using both parasternal long and short axis views on M-mode echocardiography in three cardiac cycles, as described by Iacobellis [19]. Maximum values at any site were measured and the average value was used for statistical analyses. Intraobserver agreement was tested using repeated measures and no significant differences were seen for randomly selected subjects (intraclass correlation analysis was used and the strength of the concordance was 0.94).

### Statistical methods

The Statistical Program for Social Sciences (SPSS for windows 15, Inc., Chicago, IL, USA) was used for all statistical calculations. Continuous variables are given as mean ± standard deviation; categorical variables are defined as numbers and percentages. Data were tested for normal distribution using the Kolmogorov-Smirnov test. Continuous variables were compared using the Student t test and Mann Whitney U test where appropriate. The chi-square test was used for comparison of the categorical variables between the two groups. Univariate correlation analysis was performed using the Pearson’s test to identify variables that potentially affected EAT thickness and plasma homocysteine level. Variables with a p value of <0.05 in univariate correlation analysis were included in a multivariate stepwise linear regression analysis model in order to assess the independent determinants of EAT thickness and plasma homocysteine level. All tests of significance were two-tailed. Statistical significance was defined as p <0.05.

## Results

### Study population

General characteristics of the study population are presented in Table 1. As expected, weight, waist circumference, body mass index and systolic and diastolic blood pressure were higher in the MS group. Height was not different between groups. The study group had higher plasma triglyceride levels and lower HDL levels. Plasma fasting glucose, total cholesterol and LDL cholesterol were similar in both groups.

### EAT thickness

EAT thickness was significantly higher in patients with MS (Table 1) and was similar between gender groups (5.3 ± 1.9 mm in men, 4.9 ± 1.9 mm in women, p = 0.357). In the univariate correlation analysis, body mass index, triglyceride level, weight, age and waist circumference were correlated positively whereas HDL cholesterol level was correlated negatively with EAT thickness. However, plasma homocysteine levels had the strongest correlation with EAT thickness (Table 2). Moreover, EAT thickness was found to be related to some echocardiographic measures, including ventricular septum thickness, posterior wall thickness, left ventricular mass and left atrial diameter (Table 2).

**Table 2:** Results of univariate correlation analysis for epicardial adipose tissue thickness and homocysteine levels

**Variables**	**EAT thickness**		**Plasma homocysteine level**	
	**r**	**P value**	**r**	**P value**
Plasma homocysteine level	0.584	<0.001	-	-
EAT thickness	-	-	0.584	<0.001
Body mass index	0.423	<0.001	0.261	0.001
Triglyceride level	0.383	<0.001	0.335	<0.001
Weight	0.354	<0.001	0.243	0.003
Age	0.297	<0.001	0.206	0.012
Waist circumference	0.280	0.001	0.161	0.049
HDL cholesterol level	−0.218	0.007	−0.394	<0.001
Systolic blood pressure	0.149	NS	0.352	<0.001
Diastolic blood pressure	0.076	NS	0.266	0.001
Fasting plasma glucose	0.139	NS	0.039	NS
Ventricular septum thickness	0.190	0.020	0.139	NS
Posterior wall thickness	0.249	0.002	0.156	NS
Left ventricular mass	0.219	0.007	0.126	NS
Left atrial diameter	0.267	0.001	0.148	NS

EAT = epicardial adipose tissue, NS = not significant.

### Homocysteine concentrations

Plasma homocysteine levels were higher in patients with MS (21.6 ± 6.1 μmol/L vs. 15.1 ± 5.8 μmol/L, p < 0.001) and hypertension (21.7 ± 7.3 μmol/L vs. 17.3 ± 6.3 μmol/L, p = 0.002). There was no significant difference between groups according to gender (19.2 ± 6.9 μmol/L in men, 17.8 ± 6.7 μmol/L in women, p = 0.195). EAT thickness, HDL cholesterol (reversely), systolic blood pressure, triglyceride level, diastolic blood pressure, body mass index, weight, age and weight circumference were significantly correlated with plasma homocysteine levels (Table 2). No association was found between homocysteine levels and echocardiographic measurements.

### Multivariate analyses

The multivariate stepwise linear regression analysis revealed that plasma triglyceride level, age, body mass index, left atrial diameter and diagnosis of MS were the independent determinants of EAT thickness (Table 3). The independent determinants of plasma homocysteine level were systolic blood pressure, plasma triglyceride and HDL cholesterol levels and the diagnosis of MS (Table 4). For each 1 mm increase in thickness of EAT, a 3.51 μmol/L (95% CI: 2.24-4.79) increase in plasma homocysteine level was expected.

**Table 3:** Multivariate stepwise linear regression analysis for epicardial adipose tissue

	** B**	** SRC**	** t**	** P value**	**95% CI for B**	
					**Lower bound**	**Upper bound**
Age	0.029	0.229	3.578	<0.001	0.013	0.046
Triglyceride level	0.008	0.262	3.737	<0.001	0.004	0.012
Body mass index	0.156	0.315	4.774	<0.001	0.092	0.221
Diagnosis of MS	0.741	0.197	2.734	0.007	0.205	1.278
IVS thickness	−0.028	−0.026	−0.186	0.853	−0.324	0.269
PW thickness	0.226	0.193	1.313	0.191	−0.114	0.566
LVM	−0.006	−0.165	−1.181	0.240	−0.016	0.004
Left atrial diameter	0.088	0.222	2.724	0.007	0.024	0.151

Dependent variable: Epicardial adipose tissue thickness, B = coefficient of regression, CI = confidence interval, IVS = interventricular septum, LVM = left ventricular mass, MS = Metabolic Syndrome, PW = posterior wall, SRC = standardized regression coefficient (r^2^ = 0.405).

**Table 4:** Multivariate stepwise linear regression analysis for plasma homocysteine level

**Variable**	** B**	** SRC**	** t**	** P value**	**95% CI for B**	
					**Lower bound**	**Upper bound**
HDL cholesterol level	−0.151	−0.232	−3.299	0.001	−0.241	−0.060
Diagnosis of MS	3.229	0.239	3.028	0.003	1.121	5.337
Systolic blood pressure	0.130	0.191	2.651	0.009	0.033	0.227
Triglyceride level	0.015	0.144	2.009	0.046	0.000	0.030

Dependent variable: Plasma homocysteine level, B = coefficient of regression, CI = confidence interval, EAT = epicardial adipose tissue, MS = Metabolic Syndrome, SRC = standardized regression coefficient (r^2^ = 0.404).

## Discussion

In this study, we observed that plasma homocysteine levels and EAT thickness were increased in patients with MS. MS is a combination of several disorders, in which insulin resistance and prothrombotic and proinflammatory states are common pathophysiological mechanisms [1].

Many previous studies and meta-analyses have demonstrated that EAT thickness and plasma homocysteine were increased in patients with MS and/or coronary artery disease [4,12,20,21]. Ding et al. showed that an increased quantity of EAT is associated with incident coronary artery disease and major adverse cardiac events independent of conventional risk factors [22]. There have been several meta-analyses investigating the association between homocysteine and coronary artery disease. One meta-analysis, including 72 case–control studies investigating the effect of methylentetrahydrofolate reductase gene mutation and 20 prospective studies evaluating the relation of serum homocysteine with coronary artery disease, demonstrated an odds ratio of 1.42 (95% CI 1.11-1.84) in case-controls and 1.32 (95% CI 1.19-1.45) in the prospective studies for an increase of 5 μmol/L in plasma homocysteine [23]. Similarly, a homocysteine level 25% higher than usual had an odds ratio of 1.12 (95% CI 1.04-1.20) in a meta-analysis involving 5073 cardiac events [4].

The relationship between EAT and MS is also well known. In a recent meta-analysis consisting of 2027 subjects of whom 1030 had MS, EAT thickness was significantly higher in patients with MS (standardized difference in means 1.15 mm, 95% CI 0.78 to 1.53, p =0.0001) [12]. Cut-off points for EAT thickness have been proposed to diagnose MS. Okyay et al. reported that EAT thickness of >4.35 mm indicated MS according to International Diabetes Federation criteria [21]. Although there is conflicting data regarding the association between plasma homocystene level and MS, several studies support this relationship [8-10,24,25]. In addition, in patients with coronary artery disease, plasma homocysteine levels were found to be higher in subjects with MS than in those without [10]. This association between homocysteine level and MS was also observed in patients with prior myocardial infarction [26]. However, Hajer et al. reported that while not related to increased cardiovascular risk, plasma homocystein levels were high in patients with atherosclerotic vascular disease and MS [10].

When all of these complex and entwined relations are considered, the absence of epicardial coronary artery disease in our study population distinguishes as the presence of concomitant coronary artery disease may influence the amount of plasma homocysteine level and EAT thickness. Even so, we detected significantly higher plasma homocysteine and EAT thickness in subjects with MS compared with control subjects.

EAT has various endocrine and inflammatory functions and produce mediators such as interleukin-6, interleukin-1b, tumor necrosis factor-α and monocyte chemotactic protein-1, which can cause endothelial dysfunction [27]. Unlike white adipose tissue, brown fat expresses adiponectin, which has a protective effect on endothelial function [28,29]. Several studies have shown an inverse association between brown adipose tissue and obesity, impaired glucose tolerance, and type 2 diabetes mellitus [29]. Accordingly, the dysregulated production of adiponectin may contribute to endothelial dysfunction in subjects with MS [30].

Consistent with our results, a previous study on 68 women with chest pain and angiographically normal coronary arteries revealed that EAT thickness related to microvascular dysfunction, an indicator of endothelial dysfunction [31]. Even though the authors did not measure plasma homocysteine, we may speculate that this effect is in part due to homocysteine mediated endothelial dysfunction by both inducing endothelial cell injury and impairing nitric oxide levels [5-7].

Results of our study may be interpreted as showing that plasma homocysteine and EAT thickness begin to increase before angiographically detectable coronary artery disease in patients with MS, which represent a population at high risk for coronary artery disease. Moreover, as both EAT and homocysteine lead to endothelial dysfunction, a key event in the development of atherosclerosis predating clinically obvious vascular disease [32], these two factors may contribute to the initiation, progression and acceleration of coronary artery disease in patients with MS. For this reason, plasma homocysteine level and EAT thickness may be used as additional and easy diagnostic tools for the presence of endothelial dysfunction and the necessity for follow-up for possible future overt coronary artery disease. In a recent study, Iacobellis et al. demonstrated that EAT thickness was found to be a good predictor of steatosis of non-cardiac organs such as the liver [33]. We previously showed that a relationship exists between nonalcoholic fatty liver disease and the presence of coronary artery disease [34]. These findings may also support the role of EAT on the process of coronary artery disease.

EAT volume was associated with MS and cardiometabolic risk factors, including plasma homocysteine, in patients with rheumatoid arthritis [35]. Our study also confirms the association between plasma homocysteine and EAT thickness in patients with MS. Moreover, the correlation between EAT thickness and left ventricular mass in our study is consistent with previous studies [36,37].

### Study limitations

The use of conventional coronary angiography for diagnosis of normal coronary arteries may be considered a limitation of this study. Detection of early forms of coronary artery disease (plaque formation) and better determination of the atherosclerotic process may be possible if coronary angiography had been supported with intravascular ultrasound. In addition, we did not measure additional indicators of atherosclerosis, such as carotid intima-media thickness or ankle brachial indices. Lastly, we do not have long-term follow-up data on cardiovascular events.

## Conclusions

Our study demonstrated the close relationships between MS and EAT thickness and plasma homocysteine level in the absence of overt coronary artery disease. Although we cannot reveal the underlying pathological process of these findings, we think that increased EAT and homocysteine may constitute a higher cardiovascular risk in patients with MS and can be used to help diagnosis of endothelial dysfunction in patients complaining of chest pain with normal coronary arteries.

## Abbreviations

EAT: Epicardial adipose tissue; MS: Metabolic syndrome.

## Competing interests

The authors declare that they have no competing interests.

## Authors’ contributions

Dr. ASB gathered data, performed the echocardiography and drafted the manuscript. Dr. MED helped with the drafting and interpretation of data. Dr.’s DÇ and UAB analyzed data and assisted in drafting the manuscript. Dr.’s BB and HM designed the study and helped in data interpretation. All authors contributed to the preparation and critical revision of the final manuscript. All authors read and approved the final manuscript.
